# Self-Rated Depression and Physician-Diagnosed Depression and Anxiety in Florida Adults: Behavioral Risk Factor Surveillance System, 2006

**Published:** 2008-12-15

**Authors:** Amy Z. Fan, Tara W. Strine, Ruth Jiles, Ali H. Mokdad, Youjie Huang, Melissa R. Murray, Senyoni Musingo

**Affiliations:** Centers for Disease Control and Prevention; Centers for Disease Control and Prevention, Atlanta, Georgia; Centers for Disease Control and Prevention, Atlanta, Georgia; Centers for Disease Control and Prevention, Atlanta, Georgia; Florida Department of Health, Tallahassee, Florida; Florida Department of Health, Tallahassee, Florida; Department of Children and Families, Tallahassee, Florida

## Abstract

**Introduction:**

Our purpose was to determine the prevalence and correlates of self-reported symptoms of depression and physician-diagnosed depression and anxiety in Florida adults by using the 2006 Florida Behavioral Risk Factor Surveillance System (BRFSS).

**Methods:**

The BRFSS is an ongoing, state-based telephone health survey of noninstitutionalized adults that uses random-digit dialing. In 2006, an Anxiety and Depression Module was administered in Florida. Eight questions were used to examine current depression. Two additional questions assessed health care provider diagnosis of depressive and anxiety disorders. We used SUDAAN version 9.0 to evaluate the data to accommodate the complex sampling design.

**Results:**

Approximately 9% of Florida adults experienced current depression; about 13% had had a diagnosis of depression in their lifetime and 11% had a diagnosis of anxiety in their lifetime. Approximately 44% of respondents with current depression had not had a diagnosis of depression. Current depression and lifetime diagnosis of depression and anxiety were independently associated with sociodemographic variables (being a woman, young, previously married or never married, or unemployed or unable to work), adverse health behaviors (current or former smoking, physical inactivity, or obesity), and chronic health conditions (history of a stroke, diabetes, or asthma). Although the prevalence of depression among non-Hispanic blacks and people with low education levels is higher, members of these groups are less likely than members of other sociodemographic groups to have had depression diagnosed by a physician.

**Conclusion:**

Depression and anxiety are associated with sociodemographic disadvantages and chronic conditions and risk factors. Knowing the prevalence of depression and anxiety, both self-rated and physician-diagnosed, is useful in identifying unmet mental health needs among subpopulations.

## Introduction

Mental health problems and illnesses affect 1 in 5 Americans (1,2). In fact, mental illness is the second leading cause of disability and premature mortality in the United States (1,2). Depression and anxiety, both common mental disorders, have been associated with an increased prevalence of chronic disease and unhealthy lifestyles (3-6).

Both the National Comorbidity Survey and the National Health and Nutrition Examination Survey (NHANES) provide national-level prevalence estimates of depression and other mental disorders (7,8). However, state- and local-level data are lacking and may be more helpful in planning mental health services for local populations. The Behavioral Risk Factor Surveillance System (BRFSS) is an ongoing, state-based, telephone health survey of noninstitutionalized US adults that is conducted by random-digit dialing (9,10). The BRFSS collects data on sociodemographic characteristics, chronic illness, health behaviors, access to health care, and other health-related information. Given its large sample size, the BRFSS can produce local, state, and national estimates.

BRFSS used the measure of frequent mental distress (FMD), defined as 14 or more mentally unhealthy days during the past 30 days, to represent the extent of perceived mental distress. State-level prevalence estimates of FMD have been reported previously (11). However, this measure does not provide prevalence estimates for common diagnosable mental illnesses. The Patient Health Questionnaire-8 (PHQ-8) is a well-validated, self-reported, diagnostic and severity measure of depression designed for use in primary care (12,13). Its sensitivity and specificity are comparable to those of other depression measures that are twice as long. As part of the 2006 Data Infrastructure Grants from the Substance Abuse and Mental Health Services Administration, state health departments, in collaboration with state mental health agencies and the Centers for Disease Control and Prevention (CDC), implemented an Anxiety and Depression Module (ADM) (8 of the 10 items of ADM were the PHQ-8) in 38 states, the District of Columbia, Puerto Rico, and the US Virgin Islands by using an independent probability sample of adults aged 18 years or older.

Our study is based on the data collected in Florida and is designed to determine the prevalence of self-rated depression and physician-diagnosed depression and anxiety in Florida; examine a wide array of variables associated with depression and anxiety, including sociodemographic characteristics, chronic conditions, and unhealthy behaviors; and identify the subpopulations most vulnerable to depression and anxiety but less likely to be diagnosed by physicians. The findings will improve our understanding of disparities in mental health problems and mental health services in Florida.

## Methods

### Data

The BRFSS methods, including the weighting procedure, are described elsewhere (9). All BRFSS questionnaires, data, and reports are available at www.cdc.gov/brfss.

The Florida BRFSS is a stratified random sampling survey. The sample is drawn from 3 strata defined by county population size and 1 stratum allocated for areas with at least 45% minority households. The survey's implementation follows CDC protocol. In 2006, Florida BRFSS surveyed 10,726 respondents; the survey response rate was 51.2%. Among these, 10,283 answered the questions in the ADM.

### Definition

**Self-rated current depression**

The PHQ-8 is adapted from the PHQ-9 (12,13). PHQ-9 consists of the actual 9 criteria for a diagnosis of depressive disorders, as defined in the Diagnostic and Statistical Manual of Mental Disorders, fourth edition (DSM-IV) (14). PHQ-9 has been validated among racially and ethnically diverse populations in primary care (15) and for telephone administration (16). In PHQ-8, the question assessing suicidal or self-injurious ideation from PHQ-9 was eliminated because intervention over the telephone is impossible and thoughts of self-harm are uncommon in the general population (13). Research indicated that PHQ-8 has operating characteristics similar to those of PHQ-9 and is an acceptable alternative to PHQ-9 (13).

We standardized the response set of the PHQ-8 to be similar to other BRFSS questions by asking the number of days in the previous 2 weeks the person had experienced a particular depressive symptom. This modification was approved by the original authors of PHQ-8. For analytic purposes, the modified response set can be converted back to the original response set: 0 to 1 days = "not at all," 2 to 6 days = "several days," 7 to 11 days = "more than half the days," and 12 to 14 days = "nearly every day," with 0 to 3 points assigned to the 4 categories, respectively. Item scores are summed for a total score of 0 to 24. A score from 0 to 4 represents no meaningful depressive symptoms; 5 to 9, mild depression; 10 to 14, moderate depression; 15 to 19, moderately severe depression; and 20 to 24, severe depression (12). For this study, the definition of current depression is a total score of at least 10.

**Lifetime diagnosis of depression and anxiety**

The remaining 2 questions of the ADM assess health care professional diagnoses of depressive and anxiety disorders: "Has a doctor or other health care provider EVER told you that you have an anxiety disorder (including acute stress disorder, anxiety, generalized anxiety disorder, obsessive-compulsive disorder, panic attacks, panic disorder, phobia, posttraumatic stress disorder, or social anxiety disorder)?" and "Has a doctor or other health care provider EVER told you that you have a depressive disorder (including depression, major depression, dysthymia, or minor depression)?" Possible responses include "yes," "no," "don't know/not sure," and "refused." The prevalence of lifetime diagnosis of anxiety or depression was calculated as the proportion of people who answered "yes" to the question.

**Covariates**

Sociodemographic variables included sex, race or ethnicity, age group, marital status, employment status, and health care coverage. Health care coverage was assessed by asking whether the respondent had health care coverage, including health insurance, prepaid plans such as health maintenance organizations, or government plans such as Medicare.

Unhealthy behaviors included smoking (current, former), physical inactivity (yes/no), binge drinking (yes/no), and heavy drinking (yes/no). Respondents were considered current smokers if they had smoked at least 100 cigarettes in their lifetimes and currently smoked. Former smokers were those who had smoked at least 100 cigarettes in their lifetimes and currently did not smoke. Adults were considered physically inactive if they had not participated in any leisure-time physical activity or exercise during the previous 30 days. Consistent with the 2005 dietary guidelines of the US Department of Agriculture and the US Department of Health and Human Services (17), heavy drinkers were defined as men who reported drinking more than 2 drinks per day and women who reported drinking more than 1 drink per day. Binge drinkers were defined as men who had 5 or more drinks on 1 occasion and women who had 4 or more drinks on 1 occasion during the previous 30 days.

Chronic health conditions included obesity and having been diagnosed with coronary heart disease (CHD), stroke, diabetes, or asthma. Body mass index (BMI = mass [kg] divided by height squared [m^2^]) was determined from self-reported height and weight. Adults were considered obese if their BMI was 30 kg/m^2^ or more. CHD was defined on the basis of an affirmative response to either of the following questions: "Has a doctor, nurse, or health care professional ever told you that you have had a heart attack, also called myocardial infarction?" or "Has a doctor, nurse, or health care professional ever told you that you have angina or coronary heart disease (CHD)?" Stroke was assessed by the question, "Has a doctor, nurse, or health care professional ever told you that you have had a stroke?" Diabetes status was assessed by the question, "Have you ever been told by a doctor that you have diabetes?" People who responded "yes" were considered to have diabetes. Those who responded "yes, but only during pregnancy" or "prediabetes or borderline diabetes" were considered not to have diabetes. People were considered to have asthma if they responded "yes" to the question, "Have you ever been told by a doctor, nurse, or other health professional that you had asthma?"

### Statistical analysis

We calculated the prevalence of current depression and lifetime diagnosis of depression and anxiety for groups with selected demographic and socioeconomic characteristics. The prevalence of selected chronic conditions and behavioral risk factors by depression and anxiety status was analyzed to examine the comorbidity of depression with chronic conditions and their risk factors. We used multivariate logistic regression to estimate adjusted prevalence odds ratios (APOR) and their 95% confidence intervals after adjustment for all proposed correlates of depression and anxiety. A probability less than .05 was considered statistically significant. We used SUDAAN version 9.0 (RTI International, Research Triangle Park, North Carolina) to accommodate the complex sampling design of BRFSS. The results were weighted to represent the distribution of Florida adults.

## Results

A total of 10,283 Florida adults responded to the BRFSS survey. Approximately 9% of respondents reported experiencing depression at the time of the survey. More than 13% of respondents had been diagnosed with depression by physicians and 11.2% had been diagnosed with anxiety at some point during their lifetimes. Current depression was significantly correlated with lifetime diagnosis of depression (*r* = 0.37) and anxiety (*r* = 0.31). In addition, a lifetime diagnosis of depression was significantly correlated with lifetime diagnosis of anxiety (*r* = 0.50). Approximately 82% of respondents (87% of men, 79% of women) did not have a lifetime diagnosis of depression or current depression; about 9% had a lifetime diagnosis but were not currently depressed; approximately 5% had a lifetime diagnosis and were currently depressed; and approximately 4% were currently depressed without a lifetime diagnosis. Thus, approximately 44% of the respondents with depression had not been diagnosed by physicians. Prevalence estimates of current depression and of lifetime diagnosis of depression and anxiety were examined by selected sociodemographic characteristics (Table 1).

People with depression were more often current smokers (45% vs 19%) and physically inactive (45% vs 22%) than were people without depression (Table 2). The prevalence of chronic conditions such as obesity (33% vs 21%), CHD (12% vs 7%), stroke (7% vs 3%), diabetes (14% vs 8%), and asthma (24% vs 10%) was also higher among adults with current depression. The differences in chronic conditions and unhealthy behaviors based on the status of lifetime diagnosis of depression and anxiety followed a similar pattern but with smaller differences. Most differences were significant except for binge drinking in association with lifetime diagnosis of depression and heavy drinking in association with lifetime diagnosis of anxiety (Table 2).

We performed logistic regression models with sociodemographic variables, chronic health conditions, and unhealthy behaviors as covariates. Both current depression and lifetime diagnosis of depression and anxiety were independently associated with being a woman, young, previously married or never married, unemployed or unable to work, being a current or former smoker, being physically inactive, being obese, ever having a stroke, or ever having asthma (Table 3). Neither health care coverage nor a diagnosis of CHD was significant in the multivariate models; therefore, we dropped it from the final model. Compared with non-Hispanic whites, non-Hispanic blacks and Hispanics were less likely to have a lifetime diagnosis of depression or anxiety. However, no significant difference was found between racial/ethnic groups in prevalence of current depression. A similar discrepancy also exists for education levels. People who did not complete high school were more likely to be currently depressed than those who had at least some college education. In contrast, they were not more likely or were even slightly less likely to have a lifetime diagnosis of depression or anxiety than those with at least some college education. People with a high school education were less likely to have a diagnosis of anxiety than those with at least some college, although no significant difference was detected for current depression between these 2 groups.

## Discussion

Our finding that 44% of currently depressed respondents had not obtained a diagnosis indicates that the measure of obtained lifetime diagnosis alone underestimated the actual mental health burden of adults in Florida. Our analysis provides further evidence that depression among some subpopulations is likely to be underestimated. Racial minorities and people with low education levels were less likely to report a diagnosis of depression, in spite of higher or equivalent prevalence of current depression, which indicated that they were less likely to seek professional mental health service for diagnosis and treatment.

Our study identified adults who are most vulnerable to current depression: women, young to middle-aged adults, people with less than a high school education, those previously married or never married, and those currently unemployed or unable to work. Most of the findings are consistent with previous findings (11,18,19). These adults may disproportionately make up the majority of people with diagnosable mental disorders who do not seek help or receive treatment because of the stigma associated with mental illness, lack of knowledge, or lack of adequate insurance coverage (1,2). These disparities must be considered when planning efforts to promote mental health and treat mental disorders in Florida.

Reports on the prevalence of mental health problems and their effect on different racial/ethnic groups are mixed (18). Inconsistent findings on racial differences in the rates and nature of depression are probably due to methodologic differences (7,20). The Surgeon General's report concluded that 1 in 5 Americans experience mental health problems regardless of race or ethnicity (1,2). Our study found no racial/ethnic difference in the prevalence of current depression. However, there is evidence that Americans do not share equally in the hope of recovery from mental illness. Racial and ethnic minorities collectively experience greater disability from mental illness than do whites (2), possibly because minorities receive less and poorer quality of care and not because their illness is more severe or prevalent (2).

This study indicated that depression and anxiety are independently associated with unhealthy lifestyles (eg, current or former smoking, physical inactivity) and other chronic health conditions (eg, obesity, history of a stroke, diabetes, asthma). These findings are consistent with previous BRFSS reports that used frequent mental distress as the mental health measure and other studies that used data from different sources (6,18,21-25). Although CHD was not retained in multivariate logistic regression, probably because of close correlations with other covariates (especially diabetes, stroke, and physical inactivity) in the model, it does not mean that this condition is not associated with depression or anxiety (26). As shown by the bivariate analysis (Table 2), people with a history of CHD had a higher prevalence of self-rated and physician-diagnosed depression and anxiety.

This study used a depression severity score greater than or equal to 10 to define current depression. PHQ-8 can also be used as a diagnostic screener for major depression (12). More detailed analysis can also classify a target population as having none, mild, moderate, moderately severe, or severe depression. Different treatment actions have been proposed based on the depression severity (12). The PHQ depression scale can be used not only as a surveillance tool for population prevalence estimates and trends but also for planning interventions when used in primary care and other clinical settings.

This study also demonstrated that the BRFSS is an effective source for data on the mental illness burden of a state population. However, the findings in this report are subject to some limitations. First, the information was self-reported in a telephone survey. The bias caused by inaccurate recall or self-denial cannot be assessed. Second, nearly 10% of the sample was missing 1 or more of the 8 items in the PHQ-8, so they were omitted in the estimate calculation of current depression based on the PHQ-8 summary score. The prevalence of current depression may be underestimated because those with missing data were potentially more likely to experience depression. Third, data were collected from noninstitutionalized adult residents with landline telephones and might not be generalizable to the homeless, people who use cell phones exclusively, or those residing in institutions such as residential treatment centers, jails, shelters, and hospitals, where mental disorders are more prevalent. BRFSS is planning to implement cell-phone surveys to supplement the regular landline survey. Finally, the cross-sectional nature of the survey prevents any causal inference between depression/anxiety and physical health conditions and lifestyles. Nonetheless, the findings corroborate numerous reports that document the presence of mental health disorders with chronic conditions and unhealthy lifestyles (3,22,23). Thus, amelioration of depression and anxiety may improve the prognosis of chronic diseases, and better control of chronic conditions might improve mental health.

The President's New Freedom Commission on Mental Health (27) envisioned "a future when mental illnesses can be prevented or cured, a future when mental illnesses are detected early, and a future when everyone with a mental illness at any stage of life has access to effective treatment and supports — essentials for living, working, learning, and participating fully in the community." Early mental health screening, assessment, and referral to services are not yet common practice in primary care and public health care settings. In addition, depression and anxiety are present with chronic health conditions and unhealthy lifestyles; mental health intervention should thus be an essential component of lifestyle modification in chronic care management.

The PHQ-8 is a useful tool for characterizing the burden of depression in the general population at the state level. The combination of measures of self-experience and obtained diagnosis can be used to identify unmet mental health needs in the general population. The Florida data confirm that rates of depression and other mental health disorders are increasing among both men and women in recent US birth cohorts (28,29). More efforts are needed to eliminate disparities and to increase the availability of and access to mental health services for Florida adults.

## Figures and Tables

**Table 1:** Prevalence of Current Depression^a^ and Lifetime Diagnosis of Depression^b^ and Anxiety^c^ Among Adults (N = 10,283), by Selected Sociodemographic Characteristics, Behavioral Risk Factor Surveillance System, Florida, 2006

**Characteristic**	Current Depression, % (SE)	Lifetime Diagnosis of Depression, % (SE)	Lifetime Diagnosis of Anxiety, % (SE)
**Total**	8.9 (0.5)	13.1 (0.5)	11.2 (0.5)
**Sex**			
Male	6.1 (0.8)	10.2 (0.8)	8.3 (0.7)
Female	9.5 (0.6)	15.8 (0.6)	13.9 (0.6)
**Age, y**			
18-24	13.8 (2.8)	16.0 (2.6)	10.1 (2.0)
25-34	10.0 (1.3)	12.9 (1.5)	12.0 (1.4)
35-44	8.7 (0.9)	12.7 (1.1)	13.7 (1.1)
45-54	8.7 (0.8)	16.1 (1.0)	13.2 (1.0)
55-64	6.9 (0.7)	14.9 (1.0)	11.5 (1.0)
≥65	3.5 (0.4)	8.3 (0.6)	7.2 (0.6)
**Race/ethnicity**			
White non-Hispanic	8.0 (0.6)	14.9 (0.7)	12.4 (0.6)
Black non-Hispanic	9.8 (1.7)	9.6 (1.7)	9.2 (1.7)
Hispanic	7.9 (1.0)	12.6 (0.5)	8.6 (0.9)
Other, non-Hispanic^d^	8.3 (1.9)	9.7 (1.0)	10.7 (2.0)
**Education**			
Less than high school graduate	15.0 (1.9)	13.2 (1.8)	11.9 (1.6)
High school graduate	8.6 (0.9)	13.3 (0.9)	10.7 (0.8)
Some college or higher	6.5 (0.5)	13.0 (0.6)	11.2 (0.6)
**Marital status**			
Currently married	5.4 (0.4)	10.2 (0.6)	8.7 (0.5)
Previously married^e^	11.3 (0.8)	19.0 (1.0)	15.7 (0.9)
Never married^f^	12.5 (1.7)	15.7 (1.6)	13.8 (1.5)
**Employment status**			
Currently employed	6.2 (0.6)	10.8 (0.6)	9.4 (0.6)
Currently unemployed	26.5 (3.6)	27.2 (3.8)	21.9 (3.1)
Retired	3.4 (0.5)	8.9 (0.7)	7.5 (0.6)
Unable to work	34.0 (2.8)	42.4 (3.1)	36.3 (3.0)
Homemaker or student	9.2 (1.7)	15.7 (2.1)	13.0 (1.9)
**Health plan coverage**			
Yes	7.3 (0.5)	13.0 (0.6)	11.3 (0.5)
No	11.4 (1.2)	13.4 (1.3)	10.5 (1.1)

Abbreviations: SE, standard error.

a A total score ≥10 on the Patient Health Questionnaire-8, which was standardized to be similar to other BRFSS questions by asking the number of days in the previous 2 weeks the person had experienced a particular depressive symptom. The response set was 0 to 1 days, "not at all"; 2 to 6 days, "several days"; 7 to 11 days, "more than half the days"; and 12 to 14 days, "nearly every day." Zero to 3 points were assigned to the 4 categories, respectively. Item scores were summed for a total score of 0 to 24. A score from 0 to 4 represents no meaningful depressive symptoms; 5 to 9, mild depression; 10 to 14, moderate depression; 15 to 19, moderately severe depression; and 20 to 24, severe depression (12).

b Answered "yes" to the question, "Has a doctor or other health care provider EVER told you that you have a depressive disorder (including depression, major depression, dysthymia, or minor depression)?"

c Answered "yes" to the question, "Has a doctor or other health care provider EVER told you that you have an anxiety disorder (including acute stress disorder, anxiety, generalized anxiety disorder, obsessive-compulsive disorder, panic attacks, panic disorder, phobia, posttraumatic stress disorder, or social anxiety disorder)?"

d Includes Asian, non-Hispanic; Native Hawaiian/Pacific Islander, non-Hispanic; American Indian/Alaska Native, non-Hispanic; other race, non-Hispanic; multiracial, non-Hispanic.

e Previously married includes respondents who were divorced, widowed, or separated.

f Never married includes respondents who were never married or were members of unmarried couples.

**Table 2:** Prevalence of Selected Chronic Conditions and Unhealthy Behaviors for Current Depression^a^ and Lifetime Diagnosis of Depression^b^ and Anxiety^c^, Behavioral Risk Factor Surveillance System, Florida, 2006

**Condition or Behavior^d^**	Yes, % (SE)	No, % (SE)
**Current depression (n = 9,298)**	**n = 821**	**n = 8,477**
Obesity	32.8 (2.6)	21.3 (0.7)
Coronary heart disease	11.8 (1.4)	6.7 (0.4)
Stroke	6.6 (1.1)	2.7 (0.2)
Diabetes	14.1 (1.8)	7.7 (0.4)
Asthma	23.7 (2.4)	10.4 (0.5)
Current smoker	44.5 (3.1)	19.0 (0.7)
Physical inactivity	44.5 (3.0)	22.0 (0.7)
Binge drinking	18.0 (2.7)^e^	13.7 (0.6)
Heavy drinking	7.5 (1.6)^e^	5.3 (0.4)
**Lifetime diagnosis of depression (n = 10,232)**	**n = 1,235**	**n = 9,006**
Obesity	29.3 (2.0)	21.7 (0.7)
Coronary heart disease	9.2 (1.0)^e^	7.6 (0.4)
Stroke	5.7 (0.8)	3.0 (0.2)
Diabetes	11.5 (1.3)	8.1 (0.4)
Asthma	22.3 (1.8)	10.5 (0.5)
Current smoker	34.6 (2.1)	19.2 (0.7)
Physical inactivity	32.2 (2.0)	23.9 (0.7)
Binge drinking	16.3 (1.7)^e^	13.1 (0.6)
Heavy drinking	7.6 (1.3)	4.9 (0.3)
**Lifetime diagnosis of anxiety (n = 10,241)**	**n = 1,474**	**n = 8,758**
Obesity	28.8 (1.8)	21.7 (0.7)
Coronary heart disease	10.8 (1.3)	7.4 (0.4)
Stroke	5.8 (0.8)	2.9 (0.2)
Diabetes	11.9 (1.1)	8.0 (0.4)
Asthma	22.0 (1.8)	10.3 (0.5)
Current smoker	35.4 (2.1)	18.7 (0.7)
Physical inactivity	33.1 (1.9)	23.6 (0.7)
Binge drinking	16.7 (1.8)	12.9 (0.6)
Heavy drinking	6.7 (1.1)^e^	5.0 (0.3)

Abbreviation: SE, standard error.

a The Patient Health Questionnaire-8 was standardized to be similar to other BRFSS questions by asking the number of days in the previous 2 weeks the person had experienced a particular depressive symptom. The response set was 0 to 1 days, "not at all"; 2 to 6 days, "several days"; 7 to 11 days, "more than half the days"; and 12 to 14 days, "nearly every day." Zero to 3 points were assigned to the 4 categories, respectively. Item scores were summed for a total score of 0 to 24. A score from 0 to 4 represents no significant depressive symptoms; 5 to 9, mild depression; 10 to 14, moderate depression; 15 to 19, moderately severe depression; and 20 to 24, severe depression (12). Our definition of current depression is a total score ≥10.

b Answered "yes" or "no" to the question, "Has a doctor or other health care provider EVER told you that you have a depressive disorder (including depression, major depression, dysthymia, or minor depression)?"

c Answered "yes" or "no" to the question, "Has a doctor or other health care provider EVER told you that you have an anxiety disorder (including acute stress disorder, anxiety, generalized anxiety disorder, obsessive-compulsive disorder, panic attacks, panic disorder, phobia, posttraumatic stress disorder, or social anxiety disorder)?"

d Obesity was defined as body mass index ≥30 kg/m^2^, determined from self-reported height and weight. Coronary heart disease was defined on the basis of an affirmative response to either of the following questions: "Has a doctor, nurse, or health care professional ever told you that you have had a heart attack, also called myocardial infarction?" or "Has a doctor, nurse, or health care professional ever told you that you have angina or coronary heart disease (CHD)?" Stroke was assessed by the question, "Has a doctor, nurse, or health care professional ever told you that you have had a stroke?" Diabetes status was assessed by the question, "Have you ever been told by a doctor that you have diabetes?" People who responded "yes" were considered to have diabetes. Those who responded "yes, but only during pregnancy" or "prediabetes or borderline diabetes" were considered not to have diabetes. People were considered to have asthma if they responded "yes" to the question, "Have you ever been told by a doctor, nurse, or other health professional that you had asthma?" Respondents were considered current smokers if they had smoked ≥100 cigarettes in their lifetimes and currently smoked. Former smokers were those who had smoked ≥100 cigarettes in their lifetimes and currently did not smoke. Adults were considered physically inactive if they had not participated in any leisure-time physical activity or exercise during the previous 30 days. Heavy drinkers were defined as men who reported drinking >2 drinks per day and women who reported drinking >1 drink per day. Binge drinkers were defined as men who had ≥5 drinks on 1 occasion and women who had ≥4 drinks on 1 occasion during the previous 30 days.

e The difference of prevalence of a chronic condition or an unhealthy behavior between depression/anxiety "yes" and "no" groups is not significant at *P* < .05.

**Table 3:** Likelihood of Current Depression^a^ and Lifetime Diagnosis of Depression^b^ and Anxiety^c^ Among Adults, by Demographic and Socioeconomic Characteristics, Behavioral Risk Factor Surveillance System, Florida, 2006^d^

**Characteristic**	Current Depression,^b^ APOR (95% CI)	Lifetime Diagnosis of Depression,^c^ APOR (95% CI)	Lifetime Diagnosis of Anxiety,^d^ APOR (95% CI)
**Sex**			
Male	1.00 [Reference]	1.00 [Reference]	1.00 [Reference]
Female	1.56 (1.14-2.12)	1.57 (1.27-1.95)	1.75 (1.39-2.20)
**Age, y**			
18-24	6.89 (3.43-13.85)	3.32 (1.86-5.93)	1.60 (0.86-3.01)
25-34	4.17 (2.49-7.00)	2.55 (1.72-3.78)	2.36 (1.54-3.63)
35-44	3.77 (2.33-6.09)	2.40 (1.67-3.45)	2.91 (1.98-4.27)
45-54	2.81 (1.81-4.34)	2.50 (1.81-3.45)	2.15 (1.50-3.06)
55-64	1.86 (1.26-2.76)	1.95 (1.49-2.55)	1.69 (1.26-2.28)
≥65	1.00 [Reference]	1.00 [Reference]	1.00 [Reference]
**Race/ethnicity**			
White non-Hispanic	1.00 [Reference]	1.00 [Reference]	1.00 [Reference]
Black non-Hispanic	0.72 (0.43-1.19)	0.47 (0.30-0.73)	0.59 (0.36-0.95)
Hispanic	0.82 (0.56-1.20)	0.60 (0.45-0.80)	0.72 (0.55-0.95)
Other, non-Hispanic	1.08 (0.64-1.83)	0.88 (0.54-1.42)	0.90 (0.56-1.44)
**Education**			
Less than high school graduate	1.60 (1.03-2.48)	0.74 (0.51-1.07)	0.87 (0.59-1.28)
High school graduate	0.98 (0.69-1.37)	0.83 (0.67-1.03)	0.76 (0.61-0.95)
Some college or higher	1.00 [Reference]	1.00 [Reference]	1.00 [Reference]
**Marital status**			
Currently married	1.00 [Reference]	1.00 [Reference]	1.00 [Reference]
Previously married^e^	2.01 (1.49-2.70)	1.90 (1.54-2.33)	1.74 (1.40-2.16)
Never married^f^	1.53 (1.05-2.23)	1.43 (1.02-2.02)	1.88 (1.40-2.54)
**Employment status**			
Currently employed	1.00 [Reference]	1.00 [Reference]	1.00 [Reference]
Currently unemployed	4.16 (2.54-6.81)	2.30 (1.48-3.60)	2.13 (1.42-3.20)
Retired	1.26 (0.83-1.92)	1.28 (0.96-1.72)	1.25 (0.90-1.72)
Unable to work	6.62 (4.33-10.11)	4.77 (3.39-6.69)	4.28 (2.99-6.13)
Homemaker or student	1.39 (0.79-2.44)	1.50 (1.01-2.24)	1.51 (0.99-2.31)
**Smoking status^g^**			
Never smoker	1.00 [Reference]	1.00 [Reference]	1.00 [Reference]
Current smoker	2.59 (1.87-3.60)	2.05 (1.61-2.62)	2.12 (1.65-2.71)
Former smoker	1.43 (0.94-2.16)	1.47 (1.12-1.92)	1.71 (1.27-2.31)
**Physical inactivity^h^**			
No	1.00 [Reference]	1.00 [Reference]	1.00 [Reference]
Yes	2.25 (1.71-2.97)	1.49 (1.20-1.87)	1.35 (1.07-1.70)
**Obesity^i^**			
No	1.00 [Reference]	1.00 [Reference]	1.00 [Reference]
Yes	1.65 (1.25-2.18)	1.39 (1.12-1.72)	1.40 (1.10-1.78)
**Stroke^j^**			
No	1.00 [Reference]	1.00 [Reference]	1.00 [Reference]
Yes	2.17 (1.43-3.30)	1.62 (1.11-2.37)	1.59 (1.11-2.29)
**Diabetes^k^**			
No	1.00 [Reference]	1.00 [Reference]	1.00 [Reference]
Yes	1.44 (1.00-2.09)	1.30 (0.99-1.72)	1.18 (0.84-1.67)
**Asthma^l^**			
No	1.00 [Reference]	1.00 [Reference]	1.00 [Reference]
Yes	1.63 (1.17-2.27)	1.78 (1.38-2.30)	1.81 (1.41-2.32)

Abbreviations: APOR, adjusted prevalence odds ratio; CI, confidence interval.

a The Patient Health Questionnaire-8 was standardized to be similar to other BRFSS questions by asking the number of days in the previous 2 weeks the person had experienced a particular depressive symptom. The response set was 0 to 1 days, "not at all"; 2 to 6 days, "several days"; 7 to 11 days, "more than half the days"; and 12 to 14 days, "nearly every day." Zero to 3 points were assigned to the 4 categories, respectively. Item scores are summed for a total score of 0 to 24. A score from 0 to 4 represents no significant depressive symptoms; 5 to 9, mild depression; 10 to 14, moderate depression; 15 to 19, moderately severe depression; and 20 to 24, severe depression (12). Our definition of current depression is a total score ≥10.

b Answered "yes" or "no" to the question, "Has a doctor or other health care provider EVER told you that you have a depressive disorder (including depression, major depression, dysthymia, or minor depression)?"

c Answered "yes" or "no" to the question, "Has a doctor or other health care provider EVER told you that you have an anxiety disorder (including acute stress disorder, anxiety, generalized anxiety disorder, obsessive-compulsive disorder, panic attacks, panic disorder, phobia, posttraumatic stress disorder, or social anxiety disorder)?"

d APORs and CIs were obtained after adjustment for all other variables in the table.

e Previously married includes respondents who were divorced, widowed, or separated.

f Never married includes respondents who were never married or were members of unmarried couples.

g Respondents were considered to be current smokers if they had smoked ≥100 cigarettes in their lifetimes and currently smoked. Former smokers were those who had smoked ≥100 cigarettes in their lifetimes and currently did not smoke.

h Adults were considered to be physically inactive if they had not participated in any leisure-time physical activity or exercise during the previous 30 days.

i Obesity was defined as body mass index ≥30 kg/m^2^, determined from self-reported height and weight.

j Stroke was assessed by the question, "Has a doctor, nurse, or health care professional ever told you that you have had a stroke?

k Diabetes status was assessed by the question, "Have you ever been told by a doctor that you have diabetes?" People who responded "yes" were considered to have diabetes. Those who responded "yes, but only during pregnancy" or "prediabetes or borderline diabetes" were considered not to have diabetes.

l People were considered to have asthma if they responded "yes" to the question, "Have you ever been told by a doctor, nurse, or other health professional that you had asthma?"
